# Automated Quantification of the Behaviour of Beef Cattle Exposed to Heat Load Conditions

**DOI:** 10.3390/ani13061125

**Published:** 2023-03-22

**Authors:** Musadiq Idris, Caitlin C. Gay, Ian G. Woods, Megan Sullivan, John B. Gaughan, Clive J. C. Phillips

**Affiliations:** 1 Faculty of Veterinary and Animal Sciences, The Islamia University of Bahawalpur, Punjab 63100, Pakistan; 2 School of Veterinary Science, Gatton Campus, The University of Queensland, Gatton, QLD 4343, Australia; 3 Department of Biology, Ithaca College, Ithaca, NY 14850, USA; 4 School of Agriculture and Food Sciences, Gatton Campus, The University of Queensland, Gatton, QLD 4343, Australia; 5 Institute of Veterinary Medicine and Animal Sciences, Estonian University of Life Sciences, Kreutzwalki 1, 51014 Tartu, Estonia; 6 Curtin University Sustainability Policy (CUSP) Institute, Curtin University, Perth, WA 6845, Australia

**Keywords:** behavioural quantification, cattle behaviour, digital video analysis, dietary grain content, heat stress

## Abstract

**Simple Summary:**

Cattle are vulnerable to hot environmental temperatures, and this can lead to severe heat stress, resulting in behaviour and welfare issues. The automated recording of cattle behavioural responses would be helpful in the timely diagnosis of cattle experiencing heat loading. We investigated whether video-digitised image analysis could identify behavioural responses of cattle, especially during heat stress conditions. It was further explored whether a substituted diet (in which some of the grain normally fed as a finisher diet was substituted for forage) would affect the behavioural responses to heat stress, which were measured by digitised movements. An increased digitally recorded movement in animals was observed during high environmental temperatures, which was related to stepping and grooming/scratching activities in standing animals. Under hot temperatures, cattle on the substituted diet displayed less discomfort in terms of a smaller increase in digitally recorded movements than those on the finisher diet. The results suggest that automated video digitisation software could be used as a non-invasive tool for tracking cattle behavioural responses during hot conditions and may have broader applications for behavioural studies.

**Abstract:**

Cattle change their behaviour in response to hot temperatures, including by engaging in stepping that indicates agitation. The automated recording of these responses would be helpful in the timely diagnosis of animals experiencing heat loading. Behavioural responses of beef cattle to hot environmental conditions were studied to investigate whether it was possible to assess behavioural responses by video-digitised image analysis. Open-source automated behavioural quantification software was used to record pixel changes in 13 beef cattle videorecorded in a climate-controlled chamber during exposure to a simulated typical heat event in Queensland, Australia. Increased digitised movement was observed during the heat event, which was related to stepping and grooming/scratching activities in standing animals. The 13 cattle were exposed in two cohorts, in which the first group of cattle (*n* = 6) was fed a standard finisher diet based on a high percentage of cereal grains, and the second group of cattle (*n* = 7) received a substituted diet in which 8% of the grains were replaced by lucerne hay. The second group displayed a smaller increase in digitised movements on exposure to heat than the first, suggesting less discomfort under hot conditions. The results suggest that cattle exposed to heat display increased movement that can be detected automatically by video digitisation software, and that replacing some cereal grain with forage in the diet of feedlot cattle may reduce the measured activity responses to the heat.

## 1. Introduction

Beef cattle are vulnerable to hot environmental conditions because of the high levels of nutrition, heat production and metabolism that are needed to maintain growth and, more specifically, muscle production [1,2,3,4]. During hot environmental conditions, animals may experience a deterioration in their health, productivity and welfare, as well as an increased risk of mortality [5]. The livestock industry should introduce ameliorative measures, such as reducing stocking density to avoid close contact between cattle, when there is evidence of heat stress, including, for example, panting and crowding at the water troughs [6,7]. However, improvements using these interventions could be based on a better quantification of animal responses, which would allow for early intervention [8,9]. Although alterations in performance (induced by reduced dry matter intake) and physiological changes, for example open mouth panting [2], are known indicators of heat stress in feedlots, there is a need for new automated welfare- or behaviour-based monitoring systems, especially video digitization, to assess animal responses under hot environmental conditions to improve animal welfare.

Animals alter their behaviour during hot conditions, which is linked to complex physiological changes [10,11]. The tools available for recording their behaviour vary from direct observation and/or manual recording to a completely automated locomotion recording system that uses infrared or pressure sensors or various image processing methods [12,13,14]. The automated behaviour recording systems have obvious advantages for research, as they use less labour and are less prone to bias than traditional (manual) recording systems, where observers must analyse video through direct observation and are not always blind to treatments [15]. In addition, automated recording systems do not suffer from observer fatigue, nor are there any subjective differences between the observers recording behaviours [16].

Animal applications of automated motion recording have included studying chemotaxis in worms (*C. elegans*), locomotion in the common fruit fly (*D. melanogaster*), zebra fish [17,18], pigs, broiler chickens [19,20] and a variety of birds including house finches, black-capped chickadees and a pair of northern cardinals [15]. The impact of high environmental temperatures on larval zebrafish was studied using an automated video-tracking software to quantify locomotion responses [15]. In addition to its use in a wide range of animal applications, the automated behavioural recording software has the potential to be used as a non-invasive tool to assess heat load responses in beef cattle. 

A novel application of the automated behavioural recording software PyTracker (www.github.com/enwudz/pytracker, accessed on 25 January 2020), which is based on the Python programming language and purpose-created scripts, was investigated to analyse video-digitised movement through pixels displaced by a moving object in the foreground (a Black Angus steer). The automated behavioural recording software was further tested for whether this can be used to quantify motion behaviours and indicate the presence of heat stress (and, as a result, welfare compromise). Although the Australian government has imposed constraints because of the heat stress risk in exported livestock [21], in the absence of a welfare-specific monitoring program for feedlots, it was envisaged that the PyTracker software could be used to evaluate heat stress and distress. The primary objective of this study was therefore to evaluate the movement of beef cattle exposed to hot conditions, assessed via video digitisation, and determine the association of digitised movement with cattle behaviour and other routine activities. It was further explored whether a substitution of grain with forage would affect the behavioural responses to heat stress, as measured by digitised movements.

## 2. Materials and Methods

### 2.1. Animals and Treatments

Ethical approval for the study was obtained from The University of Queensland Animal Ethics Committee (SAFS/460/16). In total, 24 yearling Black Angus steers were procured from a commercial property in Armidale, New South Wales, for a study at The University of Queensland’s Animal Science Precinct, Gatton, QLD, Australia, (27.6° S, 152.3° E) from December to April during the southern hemisphere summer. The animals had an initial non-fasted body weight of 493 ± 6.8 kg. They were randomly separated into 2 cohorts of 12 animals, which was the capacity of the climate-controlled room utilised for the study. The first cohort was fed a standard feedlot ration based on cereal grains (finisher diet), and the second received a diet in which 8% of the grains were replaced by lucerne hay (substituted diet). Only those animals that remained under camera vision for the entire observation time from each cohort were included in the study, constituting a total of 13 animals, with 6 animals fed a finisher diet (cohort 1) and 7 a substituted diet (cohort 2) during exposure to heat stress. 

Each cohort of 12 steers was kept for 50 d in a feedlot pen and then for 10 d in outdoor individual pens to acclimatise them to the handling and feeding management practices that they would receive in the climate rooms. Then, they were moved to the experimental facility where they were kept in two climate-controlled rooms (CCR) for 18 days. The animals were allowed to acclimatise to the experimental facility before the recording of actual behavioural parameters inside the climate control chambers began. They were exposed to an initial thermoneutral period (TN; day 3–4), a transition phase to hot conditions (TP1; day 5), a hot period (HOT; day 6–12), a transition phase to the recovery period (TP2; day 13) and a recovery thermoneutral period (Recovery; day 14–17) (Table 1). During the TN and Recovery periods, the ambient dry bulb temperature (T_A_) and relative humidity (RH) in the CCR was maintained at 20 °C and 65%, respectively. During the HOT period, the T_A_ and RH increased each day from 07:00 h to reach a maximum at 11:00 h, which was maintained until 16:00 h and then decreased hourly from 16:00 h to reach the daily minimum T_A_ and RH at 20:00 h. The minimum T_A_ declined over the HOT period before cattle entered the Recovery period.

### 2.2. Animal Facilities

In the initial feedlot phase, animals in each cohort were kept in a feedlot pen of 162 m^2^ (27 m × 6 m) with an east–west alignment. Each contained a concrete feed bunk, water troughs and a shaded area of 1.3 m^2^/animal at midday. In the next phase, animals were randomly assigned to individual pens in an outdoor facility, and then to individual pens in two climate-controlled rooms. The outdoor pens and CCR facility have been described in detail by Sullivan et al. [22]. The ambient dry bulb temperature, humidity cycles and lighting schedule in each room were programmed automatically. The ventilation rate inside each CCR was maintained with a centrally controlled air-conditioning system. Oxygen, ammonia and carbon dioxide were monitored at 06:00, 12:00 and 18:00 h (and at midnight on hot days) each day, using a hand-held gas meter (Gas Micro Alert, BW technologies, Honeywell). Lighting was set at 10% of the maximum from 19:00–05:00 h, and was maximum at all other times. The individual cattle pens (2.5 × 2.5 m) each had rubber mat flooring over a steel grill, which facilitated the drainage and cleaning of the pens. Pens were cleaned daily at 06:30–07:30 h, prior to feeding at 09:00 h, by hosing all excrement from the mats and pen flooring. All animals were provided with individual access to a water trough and a feed trough (500 × 500 × 500 mm). The climate-controlled facility was provided with cameras (K-guard CW214H; New Taipei, China), with two cameras over each pen attached to a digital video recorder (LG, XQ-L900H; Seoul, Republic of Korea) for surveillance of the animals.

### 2.3. Animal Management

The immunisation of animals followed a similar regime as that outlined by Sullivan et al. [22]. Animals were injected with a hormonal growth promoter (HGP) implant (Synovex^®^ containing trenbolone acetate, Zoetis, Parsippany, NJ, USA) upon entry to the feedlot.

Upon entry to the feedlot, steers in cohort 1 were offered a starter diet of concentrate only for the first 8 days, then an intermediate diet for 6 days, and then were transitioned to a finisher diet over the next 3 days, which they were fed until the end of the trial (Table 2). Due to an adverse heat stress response of some cattle in the first cohort, the second cohort was fed an alternative diet from the second day of the hot period and were transitioned back to the finisher diet over four days during the recovery thermoneutral period. Individually housed cattle were fed their diet at 2.5% of their body weight on a DM basis, with refusals removed and weighed each morning prior to the provision of 50% of the ration at 09:00 h and the remainder at 13:00 h. Feed dry matter content was determined by oven drying. The animals were provided with ad libitum water during the study, and water consumption in the CCR was recorded at the time of each observation using water meters (RMC Zenner, Eagle Farm, QLD, Australia). 

### 2.4. Automated Behavioural Quantification and Other Key Observations

There was 24 h camera surveillance of all individually penned cattle using two cameras at the front and back of each pen. Quantification of the animal movement from the recorded videos was obtained using custom built Python-based automated video-tracking software (www.github.com/enwudz/pytracker, accessed on 25 January 2020). Different components of the software were installed as described by Conklin et al. [15]. The automated behavioural quantification of the animals’ movement inside the climate-controlled rooms was observed continuously for a 5 min period each hour for day 3 (TN); 6, 8, 10 and 12 (HOT); and 16 (Recovery) from videos recorded over 24 h.

Secondly, to investigate the association of the digitised movement measures with recorded behaviours, a 60 s length of a video clip for each animal (*n* = 13) was analysed (total of 39 video clips) on d 3 (TN), 6 (HOT) and 15 (Recovery). The behaviours most relevant to the heat stress were selected for this study. From the video recordings (60 s), digitised movement (Python based video-tracking software), as well as other key behaviours such as standing, lying, stepping of all four limbs, eating, ruminating, and grooming and scratching, were continuously recorded using the behaviour-coding software BORIS v. 6.0.4 [23] for a 1 min duration each day for each animal on day 3 (TN), 6 (HOT) and 15 (Recovery) (Table 3). 

The selected behaviours were among the observable behaviours that could be easily seen from a camera position above the experimental animals. Open mouth breathing is a major behavioural indicator of heat stress in cattle [25]. Since it is not observable from above the animal, it was not included in the ethogram. During analysis, video clips with motion artefacts such as glare, moving shadows, changes in light intensity or any distortion in the video image, as well as video clips showing the presence of people near the pens, were excluded from the analysis.

### 2.5. Climatic Data

The climate inside the climate-controlled room was maintained using a cyclic air-conditioning system to maintain ambient temperature and humidity. Climatic conditions (T_A_ and RH) inside each CCR were monitored at 10 min intervals using two temperature and humidity data loggers per room (HOBO UX100-011, Onset, MA, USA) that were installed on the pen 1.5 m from the ground. A temperature humidity index (THI) was calculated using the following equation, adapted from Thom [26]:

THI = (0.8 × T_A_) + [{(RH/100) × (T_A_ − 14.4)} + 46.4] (1)

where RH = relative humidity in % and T_A_ = ambient temperature in °C.

### 2.6. Statistical Analyses

As only those animals that remained under camera vision for the entire observation time from each cohort were included in the study, the data obtained from 13 steers (6 animals from the finisher diet cohort and 7 from the substituted (grain substituted with forage) dietary cohort were analysed by using the statistical software Minitab 18 (Minitab^®^ 18.1 Inc. Chicago, IL, USA) for Windows.

The video-digitised movement of animals was analysed using a mixed effects model with the following fixed factors: cohorts (D; finisher and substituted diet), treatment period (P; HOT and Recovery), day (d) of the experiment nested within the treatment, and the interactions diet x treatment and diet x day. The animal identification (ID) was included as a random factor. Data from the TN period were used as a covariate (Cov). The equation for the analysis is:

Y_VDM/5 min_ = µ + D + P + d (P) + ID + (D × P) + (D X d (P)) + Cov + e (2)

where Y_VDM_ is the expected value for movement response (video-digitised) variables; μ is the expected mean value for response variables equal to zero, where the factors are as described above; and e is the random error associated with experimental observations. The changes in the digitised movement (pixel changes/5 min) in feedlot cattle during the high-temperature treatment (HOT) and TN periods were analysed using a mixed effects model with the following fixed factors: cohorts (D; finisher and substituted diets during the HOT period), treatment period (P; HOT and TN), animal standing or lying (S/L), and the interactions diet × treatment and P × S/L. The animal identification (ID) was included as a random factor.

Means for each animal’s pixel displacement (pixel changes/min) for standing and lying cattle on each day were used to investigate the linear relationships between behavioural responses of cattle via a mixed effects model that included the following fixed factors: cohorts (P; HOT and Recovery), standing and lying cattle (S/L), P × S/L and the animal identification (ID) as a random factor. Additionally, values recorded from the TN period were included as a covariate in the model.

Y_VDM/min_ = µ + P + S/L + (P X S/L) + ID + Cov + e (3)

where Y_VDM/min_ is the expected value for movement response (video-digitised movement of standing or lying cattle in one min) variables; μ is the expected mean value for response variables equal to zero; and e is the random error associated with experimental observations. The changes in the digitised movement (pixel changes/min) of standing/lying feedlot cattle during HOT and TN periods was also analysed using a mixed effects model with the same random and fixed factors, as well as the interactions described above. Logarithmic transformations (Log_10_+1) were made when necessary to satisfy the Kolmogorov–Smirnov test for the normal distribution of residuals. Minitab 18 (Minitab^®^ 18 Inc.) for Windows was used for all analyses. 

The association of video-digitised movement of standing animals during the 60 s periods based on the stepping of each limb, eating, ruminating and grooming/scratching was analysed using stepwise regression (α to enter variables = 0.15) in a general linear model (GLM).

## 3. Results

**Figure 1:**
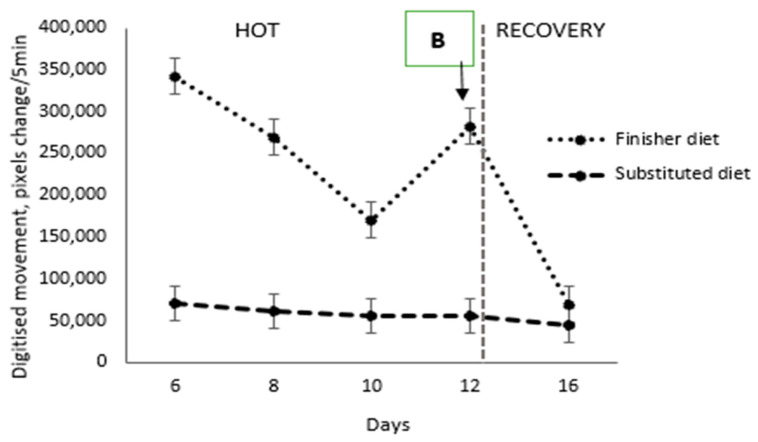
The daily video-digitised movement (pixel changes/5 min) in finisher and substituted diet cohorts of feedlot cattle, with indication of timing of the biopsy (B).

The video-digitised movement in terms of pixel displacement of the feedlot cattle (*n* = 13) exposed to high temperatures (HOT) and during TN or Recovery periods are presented in Table 4 and Table 5, respectively. The digitised movement of cattle was greater in the HOT period than in both TN and Recovery periods. Digitised movement was greater for cattle receiving the finisher diet than the substituted (grain substituted with forage) diet when in the HOT period, and there was no difference in the Recovery period when both cohorts were on same diet on day 16 (Figure 1). Day 12 was an exception, when a sudden increase in the digitised movement may have been due to a muscle biopsy being taken on that day.

The video-digitised movement, measured as pixel displacement, was greater in standing animals than in lying cattle, with no significant differences observed between periods or in the interactions with the periods (Table 4 and Table 5). 

The stepwise regression analysis found that the digitised movement in standing animals was correlated with two variables, back left limb stepping and grooming/scratching:

Digitised movements in standing animals (pixel changes per minute) = −655,030 (±470,921; *p* = 0.17) + 280,318 back left limb stepping behaviours (counts per minute) (±68,624; *p* ≤ 0.001; F_value = 16.69) + 5,640,704 groom or scratch behaviours (prop. of time) (±1,075,470; *p* ≤ 0.001; F_value = 27.51; r^2^_adj = 54.96%) (Equation (4)).

## 4. Discussion

The impact of hot conditions on behavioural responses of feedlot cattle was studied in order to investigate whether discomfort, as measured by pixel displacement, in beef cattle would be increased by hot conditions, which was assessed via video digitisation. A secondary hypothesis was that the digitised movement in feedlot cattle would be associated with specific cattle behaviours and other routine activities. It was also investigated whether a substituted diet (grain substituted with forage) would affect the behavioural responses to heat stress, which was also measured by digitised movements. The different hypotheses are discussed separately.

An automated locomotion recording system based on Python software has already been used for monitoring video-digitised movement in larval zebrafish, house finches, black-capped chickadees and a pair of northern cardinals [15], and the current study aimed to use it to examine the automated recording of behavioural responses of feedlot cattle to a heat load condition. In the current study, there was an overall increase in digitised movement in terms of pixel displacement in feedlot cattle in the HOT period as compared with the TN and Recovery periods. Increased digitised movement in terms of pixel displacement responses have been reported in larval zebrafish exposed to a high-temperature (36 °C) treatment when using automated video-tracking software [15]. Cattle express discomfort to heat load conditions through increased time spent in a standing position, increased panting and an increased respiration rate [27,28,29]. In the current study, the increased digitised movements in the cattle during the HOT period can be attributed to the pixel displacement associated with the animal’s efforts to cope with hot conditions.

A high-fibre diet is associated with increased heat production due to increased acetate production, which is relative to glucose and propionate, from microbial fermentation [30,31]. This increased potential of the high-fibre diet to elevate heat production puts livestock at risk of heat stress [32]. An increased supply of dietary energy and protein, required for maximal growth, may mean that the essential fibre requirements for optimal rumen functionality are not achieved [33,34]. Adequate fibre is necessary to maintain a stable and high rumen pH, which it achieves by stimulating saliva production that its pH buffers during the chewing of boluses in rumination [35]. Cows fed a high-concentrate diet during hot environment conditions typically have a low rumen pH [36,37], which increases the risk of severe stress responses in animals exposed to hot environment conditions [38,39]. In severe cases, rumen acidosis may result in laminitis, accompanied by ataxia, blindness and incoordination in standing animals [33,34,40]. It is therefore likely that the greater digitised movements in the finisher dietary cohort is attributable to a severe stress response due to digestive disturbances in animals on the comparatively low-fibre diet. This paper mainly focussed on the behavioural responses of heat stressed cattle; for further details of the responses to the two diets, the reader may refer to Idris [24].

The provision of an adequate opportunity for lying and standing is considered important for the maximum production, comfort and welfare of cattle [41,42]. An increase in standing time and decreased lying time has been noted previously to be due to hot environmental conditions [27,29,43,44] and poor housing [41], and it has been associated with discomfort in cattle [45]. Increased standing provides an opportunity for the dissipation of accumulated heat through evaporation and convective heat exchange [46]. Considering the importance of standing and lying behaviour in cattle, the association of video-digitised movement with standing and lying behaviours was expected. The lying behaviour depicts resting, and standing is associated with active animals performing different activities [41]. The greater digitised movement response in standing animals as compared with lying animals in the current study supports the hypothesis that digitised recordings can detect increased cattle movements.

Further, the increased video-digitised movement in terms of pixel displacement for standing cattle was associated with left back limb stepping and grooming/scratching behaviours. The back left limb of sheep responds more to the stress from floor movement than the back right limb, which appears to act as a pivot [47]. The significance of the left limb is presumed to relate to its connection to the right brain hemisphere, which controls stress responses. Cattle spent much of the rest of their time performing various routine activities, especially grooming and scratching [48]. Stepping behaviour plays a major role in adjusting body position to maintain body balance and in expressing discomfort in various stressful conditions, such as transportation stress [49], responses to novel stimuli [50,51], hoof lesions [52] and/or painful responses [53]. The association of video-digitised movement with stepping and grooming/scratching activities reflects the ability of the software to track key animal movements. Despite the unexpected reduction in sample size, the results from this experiment are statistically significant and support the hypothesis that automated video-digitised software has the potential to estimate behavioural changes in feedlot cattle. 

In hot environmental conditions, increased panting and standing has been reported in agitated cattle to dissipate accumulated heat from the body [44,45]. There is a possibility that this increased respiratory effort may contribute to body movements and should be considered as a possible factor in further experiments. Further testing is required to determine the specificity and sensitivity of this software for heat stress detection through the inclusion of other heat stress-related behaviours that contribute to movement responses, and thus its potential use in feedlots as a means of real-time detection can be further developed. 

### Limitations of the Study

The main limitation of this experiment was the small number of animals (*n* = 13), a subset of the total number of animals employed in the trial (*n* = 24), that were selected to observe video-digitised movement in heat-stressed animals. The short duration of the study could also be considered a limitation of the trial; however, this was meant to simulate a typical heat wave in Australia. This study had a relatively small number of behaviours that were recorded, primarily because of the large amount of video footage that needed coding. Later studies can investigate other detailed behavioural patterns. This could include respiratory movements of the chest and mouth, but in this study, observing respiratory movements with the camera positioned sufficiently far away from the animal to include the full torso was not possible as the chest movements were too subtle.

## 5. Conclusions

The increased digitised movement in animals following an increase in environmental temperature in the current study supports the hypothesis of increased pixel displacement in feedlot cattle during hot environmental conditions. Cattle on the substituted diet with relatively high amount of forage and lower amount of grains coped better with the heat than those on a finisher diet, displaying fewer digitised movements. Those on the finisher diet with greater digitised movements likely expressed discomfort during hot conditions. Increased digitised movements in standing cattle and the existence of an association with stepping and grooming/scratching activities reflect the usefulness of automated behavioural quantification software for future applications in the feedlot industry. It is concluded that automated video digitisation software can be applied as a useful tool for tracking cattle movements and behavioural responses during hot conditions and may have broader applications for behavioural studies.

## Figures and Tables

**Table 1:** The ambient temperature, relative humidity and temperature humidity index for finisher and substituted diet cohorts of cattle when in the climate control facility.

Day	Treatment Phase	Min T_A_ (°C)	Max T_A_ (°C)	Mean T_A_ (°C)	Min RH (%)	Max RH (%)	Mean RH (%)	Min THI	Max THI	Mean THI
0	ACC	19.7	21.0	20.1	60.9	90.6	66.2	65.5	69.1	66.3
1	ACC	19.7	21.0	20.1	60.9	90.6	66.2	65.5	69.1	66.3
2	ACC	19.5	21.6	20.0	60.0	89.3	67.1	65.3	68.9	66.2
3	TN	19.5	20.8	19.9	61.3	90.1	67.9	65.3	68.8	66.1
4	TN	19.6	24.0	20.2	60.0	89.1	68.2	65.5	72.0	66.4
5	TP1	19.9	40.5	33.2	42.9	88.4	66.1	66.3	92.6	84.9
Finisher Dietary Cohort—Transition to 30 °C from 00.00 h on day 5 Substituted Dietary Cohort—Transition to 30 °C from 21.00 h on day 5										
6	HOT	28.4	40.2	33.0	43.3	82.8	65.8	80.5	91.8	84.8
7	HOT	28.4	38.1	32.1	42.3	84.2	63.7	78.3	89.1	83.0
8	HOT	24.9	34.3	28.7	44.3	82.0	65.9	73.6	85.0	78.4
9	HOT	22.6	34.4	28.0	45.81	79.5	66.2	69.9	85.7	77.5
10	HOT	20.6	30.3	24.3	54.4	80.5	66.7	67.2	80.0	72.3
11	HOT	20.4	30.4	24.2	45.3	80.6	65.8	67.1	79.2	72.0
12	HOT	19.7	21.3	20.3	50.0	90.5	64.6	65.8	68.8	66.4
13	TP2	19.7	20.7	20.1	56.4	91.3	65.5	65.6	68.6	66.2
14	Recovery	19.7	21.4	20.1	58.1	89.0	66.7	65.6	69.6	66.2
15	Recovery	19.6	20.5	19.9	58.4	90.3	66.4	65.6	68.2	66.0
16	Recovery	19.4	25.0	20.5	57.8	93.5	66.4	65.2	73.2	66.8
17	Recovery	19.3	23.7	21.1	58.1	69.0	61.9	64.9	71.1	67.5

T_A_: ambient temperature (°C); RH: relative humidity; THI: temperature humidity index; ACC: acclimatisation to climate-controlled facility; TN: thermoneutral conditions before high-temperature treatment; TP1 and TP2: transition phases to and from hot conditions, respectively; HOT: high-temperature treatment; Recovery: thermoneutral conditions after high-temperature treatment as a recovery period.

**Table 2:** Diet ingredients and nutrient composition for starter, intermediate, finisher and substituted diets fed to cattle.

Item	Starter	Intermediate	Finisher Diet	Substituted Diet
Ingredients, % of diet				
Grain mix *	62.1	74.5	86.8	78.7
Whole cottonseed	9.0	16.5	9.0	9.0
Lucerne hay	28.9	9.0	4.2	12.3
Nutrient composition				
DM, g/kg fresh weight	880	893	887	886
ADF, g/kg DM	263	257	119	177
NDF, g/kg DM	404	375	229	253
NE_g_, MJ/kg DM	29	29	30	30
ME, MJ/kg DM	116	119	132	131
DE, MJ/kg DM	143	147	163	162
Crude fibre, g/kg DM	218	197	87	124
Nitrogen-free extract, g/kg DM	503	548	678	685
Fat, g/kg DM	46	43	46	43
Feed digestibility, g/kg DM	768	791	861	868
Digestible DM, g/kg DM	676	707	763	769
Digestible protein g/kg DM	133	125	130	131
Starch, g/kg DM	229	218	432	432

* Grain mix: feedlot pellet, 9.2%; steam rolled barley, 89.2%; vegetable oil, 1.6%. The feedlot pellet contained milled wheat, 55.9%; ammonium sulphate, 2.6%; rolled wheat, 12.5%; calcium carbonate, 15.6%; Rumensin 100, 0.3%; magnesium oxide, 0.7%; zinc supplement (Availa zinc 100), 0.34%; vegetable oil, 3.1%; NaCl, 2.8%; urea, 5.7%; vitamin A 500, 0.009%; vitamin E, 0.057%; mineral supplement (XFE-Select L), 0.385%.

**Table 3:** Ethogram for recorded behaviours for cattle housed in individual pens in the climate-controlled facility.

Item	Description
Standing	Animal standing with limb positioned upright
Lying	Animal resting on the floor with their limb laterally or sternally recumbent
Eating	Animal consuming feed at the trough
Rumination	Animal chewing the cud or regurgitating bolus
Grooming/Scratch	Animal licking any part of the body or striking one part with another part of the body or with fixture of the pen
Stepping	
Front right (FR) limb	Animal raising a front right limb and replacing it forthwith on the surface of pen
Front left (FL) limb	Animal raising a front left limb and replacing it forthwith on the surface of pen
Back right (BR) limb	Animal raising a back right limb and replacing it forthwith on the surface of pen
Back left (BL) limb	Animal raising a back left limb and replacing it forthwith on the surface of pen

Adapted from Idris [24].

**Table 4:** Video-digitised movement of cattle (*n* = 13) receiving a finisher or substituted diet and exposed to high temperatures (HOT) or initial thermoneutral period (TN).

Behaviour	Periods		SED	f-Value (d.f. ^†^)	*p*-Value			
	TN	HOT			Period (*p*)	D × P	S/L	P × S/L
Video-digitised movement, Log_10_+1 (pixel changes/5 min)	4.95 ^b^ (89,579)	5.12 ^a^ (131,940)	0.0684	7.56 (1, 21)	0.012	0.29	-	-
Video-digitised movement of standing and lying cattle, Log_10_+1 (pixel changes/min)	5.27 (184,926)	5.40 (248,312)	0.223	0.83 (1, 35.69)	0.37	-	≤0.001	0.85

Log_10_+1: log to the base 10 + 1; SED: standard error of the difference between two means; HOT: high-temperature treatment period on day 6, 8, 10 and 12; TN: thermoneutral period (Day 3) before high-temperature treatment; ^†^ treatment: error degrees of freedom; D: diet; P: period; D × P: diet x period; P × S/L: period × standing/lying. Means with different superscripts differ significantly *p* ≥ 0.05 by Fisher pairwise comparisons.

**Table 5:** Video-digitised movement of cattle (*n* = 13) receiving a finisher or substituted diet and exposed to high temperatures (HOT) or a thermoneutral recovery period.

	Period		SED	f-Value (d.f.^†^)	*p*-Value					
Parameters	HOT	Recovery			Period (P)	Diet (D)	D × P	D × d	S/L	P × S/L
Video-digitised movement (pixel changes/5 min)	163,112 ^a^	56,814 ^b^	28,426	44.48 (1, 54)	≤0.001	≤0.001	≤0.001	0.003	-	-
Video-digitised movement of standing and lying cattle, Log_10_+1; pixel changes/min (pixels change/min)	5.37 (235,012)	5.10 (126,209)	0.287	3.13 (1, 32)	0.086	-	-	-	≤0.001	0.241

Log_10_+1: logbase 10 + 1; SED: standard error of the difference between two means; HOT: high-temperature treatment period on day 6, 8, 10 and 12; Recovery: thermoneutral period after high-temperature treatment on day 16; ^†^ treatment: error degrees of freedom; D: diet; d: day; P: period; D × P: diet × period; D × d: diet × day; S/L: standing or lying; P × S/L: period × standing/lying. Means with different superscripts differ significantly *p* ≥ 0.05 by Fisher pairwise comparisons.

## Data Availability

Not applicable.
